# Supplementary material for: Expression profile of sonic hedgehog signaling-related molecules in basal cell carcinoma
Source: PLoS One. 2019 Nov 22;14(11):e0225511. doi: 10.1371/journal.pone.0225511 (PMC6874381; doi:10.1371/journal.pone.0225511)
Supplement: Attachment — Submitted filename: PONE-D-19-24006 revision comments.docx [file pone.0225511.s001.docx]

The manuscript reveals compelling evidence about mRNA expression levels of Hedgehog pathway components (GLI1, GLI2, PTCH1, PTCH2, SHH and SMO) in different skin tumors and the expression profile observed was compared to stem cells markers (LGR4, LGR5, LGR6, and LRIG1) content. Also, protein expression and subcellular localization was studied by IHC technique. In this setting, Gli1 was found overexpressed in BCC at mRNA and protein levels.

- In fact, some discrepancy was shown between both RNA and protein expression pattern in the other HH components. This is particularly important in GLI1 and GLI2, since both proteins can be found as activator (full-length) as well as repressor (proteolytically cleaved) forms. The authors have to take this consideration when interpret and discuss your IHC results.

- Additionally, the authors not found increased SMO mRNA level in BCC, when have been recently reported increases in this messenger in BCCs from Gorlin syndrome, even after adjustment by germinal and somatic mutation carrier status (Martinez MF; Cells, 2019). This is also interesting to include when this particular issue is discussed.
- The correlation analysis between HH-related molecules and stem cells markers results challenging to this reviewer:
- . In **result** text (page 8, lines 2-5), the correlation scores and P values for GLI1 probably correspond to the GLI2 (see Figure 6A and 7). Please, check the data and correct both values and its interpretation.
- . Why the correlation of SMO and SHH mRNA levels with LGR4, LGR5, LGR6, and LRIG1 are missing? This issue need to be solved to understand overall relationship between HH pathway and stem cells markers.
- Some bars in the graphs, such as the EP in **Figure 2E** and the GLI IHC of **Figure 5B**, have no dispersion. Check carefully the data presentation throughout the manuscript.
  - The **figure 6B** are confusing about the methodology used:
- . If the authors try to show the *in situ* hybridization of GLI1 and LRG5, no mention concerning GLI1 in methods were found.
- . If the authors try to show IHC of GLI1 and LRG5 proteins, the entire legend of figure 6 need to be rewritten to clearly state which variable is represented in each case.
  - Would important to add a comment in **Introduction** about the reason/s to study the stem cell markers and its relationship with HH pathway.
  - The amount of samples studied are confusing. In the first paragraph of **subjects** section of **Materials and methods** stated “…152 formalin-fixed, paraffin-embedded (FFPE) human skin tissues (normal skin, n = 5; skin tumors, n = 147)…” but in the next sentence and in **Tissue microarray construction** declared 168 skin tumors. Is important to correct and uniform the number of samples studied. In addition, since the clinicopathological features of patients are not analyzed, its mention in the text is unnecessary.
  - The number of samples of each subtype of BCC have to be included in figure 5B.
  - The final sentence of Discussion point out the relationship between HH and Wnt signaling in BCCs, but this affirmation are not supported by the results. Then, would more appropriate a suggestion of link between both pathways.

Minor changes:

- Second paragraph of **Introduction**, line 2: “…12transmembrane receptor for SHH that has a regulatory effect on the pathway (8). in the absence of…” replace by “…12 transmembrane receptor for SHH that has a regulatory effect on the pathway (8). In the absence of…”
- In page 5, line 4 replace Anti-PTCH2 by anti-PCTH2.
- In page 6, last paragraph, include HDA samples in “SHH expression was slightly higher in several tumors including BCC, TE, EP, and SPA (Fig. 1E)”
- In page 7, close de parenthesis in “…PMC (n = 13)…”
- In page 8, line 5, replace PTC2 by PTCH2.
