# Supplementary material for: Expression profile of sonic hedgehog signaling-related molecules in basal cell carcinoma
Source: PLoS One. 2019 Nov 22;14(11):e0225511. doi: 10.1371/journal.pone.0225511 (PMC6874381; doi:10.1371/journal.pone.0225511)
Supplement: Attachment — Submitted filename: Rebuttal letter.doc.docx [file pone.0225511.s003.docx]

**We appreciate the careful evaluation of our manuscript and revised our manuscript to address the issue raised by reviewer and editor.**

1. The revised version of the manuscript has been carefully checked and the author’s responses are satisfactory and greatly improve the overall quality of the paper. In the view of this version, only one issue has to be corrected:

In page 8, lanes 7-8, “… GLI1 expression showed a strong positive correlation with LGR4 (r^2^=0.62, P < 0.001), LGR5 (r^2^=0.60, P < 0.001), and LGR6 7 (r^2^=0.21, P = 0.05), but not with LRIG1…” needs to be rewrite as “… GLI1 expression showed a strong positive correlation with LGR4 (r^2^=0.62, P < 0.001), LGR5 (r^2^=0.60, P < 0.001), **but not with** LGR6 (r^2^=0.21, P **=** 0.05) **and** LRIG1…” . As is stated in statistical analysis section, “A P-value **<** 0.05 was considered statistically significant”, both LGR6 and LRIG1 have to be considered not significant.

: As suggested, the sentence has been rewritten in the revised manuscript.

2. We carefully checked out the manuscript to make sure that a gene/RNA is is in italics and protein names are not italicized.
